# AD|ARC (Administrative Data| Agricultural Research Collection): Linking individual, household and farm business data for agricultural research – The challenges and opportunities of partnership working across ADR UK

**DOI:** 10.23889/ijpds.v8i2.2238

**Published:** 2023-09-14

**Authors:** Nicholas Webster, Jen Hampton, Scott McFarlane, Peter Wilgar

**Affiliations:** 1 Welsh Government, Cardiff, United Kingdom; 2 ONS, Newport, United Kingdom; 3 Scottish Government, Edinburgh, United Kingdom; 4 NISRA, Belfast, United Kingdom

## Objectives

ADARC is a partnership of six government departments and three research institutions across ADR-UK aiming to build four Research Ready Datasets about farming households. A|ARC requires government data linking experts to work closely with agricultural statisticians and academics across traditional boundaries pooling a wide range of data and expertise.

## Methods

The ADARC partnership aims to build a model of collaborative working for administrative data research. ADARC brings together those collecting data with those linking and analysing de-identified data and eventually those using results to develop future policies. The data is from several different sources at individual, household and business levels requiring new methodologies developed by this collaboration. ADARC is also working between all four ADR centres to share learning across the ADR network building relationships for future projects beyond agriculture. The linkage process has been different in all four nations but this has offered new learning for all involved.

## Results

Due to the Covid-19 pandemic, Phase 1 of the project has been slower than hoped. However, ADARC has shown that it is possible to conduct a truly UK-wide four nation project. Data linking is underway in all four nations bringing together data on individuals, households and businesses and analysis has begun in Wales and England. The project to date has faced the usual challenges of data acquisition and data quality issues which the partnership approach has been vital for overcoming. Despite this, we’ve demonstrated that there’s a huge level of enthusiasm for working together across organisational barriers. ADARC has just been funded for Phase 2 to expand the datasets in scope and explore the feasibility of linking in spatial data.

## Conclusion

ADARC has created a model which demonstrates that partnership working is essential for fully achieving the objective of ADR UK of unlocking the potential of administrative data. The lessons of ADARC also provides a powerful case study for future cross-UK projects far beyond agriculture.

